# Morality and Marriage

**Published:** 1920-11-27

**Authors:** 


### Morality and Marriage.

The largely attended conference of social workers which has been held at Newcastle to discuss various aspects of the question of public morals, was made specially interesting by the outspoken remarks of the Dean of Lincoln (Dr. T. C. Fry). In calling attention to disastrous effects on the race of the dissemination of venereal disease, the Dean boldly announced himself in favour of the compulsory production of a health certificate before marriage. It sounded like a strong advance, he added, but he was certain that neither of his two sons would raise the slightest objection of giving a certificate of his own health to the father of a bride. Another clergyman went even further than this, and suggested that legislation should be secured to make it illegal for any minister of religion to perform a marriage ceremony without the production of a certificate of health relating to both parties. The statements made at this conference furnish one of many indications that public opinion is rapidly ripening in favour of a great safeguard of the public health against which there was until quite recently an instinctive and almost universal attitude of dread and dislike.

There was a good deal of plain speaking at this conference. Dr. E. Turner, who has just returned from a moral and medical education campaign to the Army of Occupation in Germany, powerfully advocated the importance of an early and positive education in the facts of life; and Miss Alison Neilans, secretary of the Association for Moral and Social Hygiene, put forward the view that, while the single standard of morality for both men and women is coming, it is coming the wrong way. Women, she says, are dropping down to men's standard, though she does not think it is a stage that will last long, and she is convinced that it is unnecessary to have a double standard. She was on more controversial ground in declaring that prostitution is not due to economic conditions. "Women," she asserts, "enter very largely into that kind of life because they are weak-willed; because they are in the habit of taking the line of least resistance; and because they are lazy and not inclined to work, and can get money easily in that way." It is a sweeping dictum, but it has the support of some previous writers on this subject.

